# Tunable Luminescence and Energy Transfer of Sr_3_B_2_O_6_:Ce^3+^, Sm^3+^ Phosphors with Potential Anti-Counterfeiting Applications

**DOI:** 10.3390/ma15155189

**Published:** 2022-07-26

**Authors:** Yiyi Ou, Junyu Wei, Hongbin Liang

**Affiliations:** MOE Key Laboratory of Bioinorganic and Synthetic Chemistry, KLGHEI of Environment and Energy Chemistry, School of Chemistry, Sun Yat-sen University, Guangzhou 510006, China; ouyy35@mail2.sysu.edu.cn (Y.O.); weijy35@mail2.sysu.edu.cn (J.W.)

**Keywords:** Sm^3+^, Ce^3+^, tunable luminescence, energy transfer, optical anti-counterfeiting

## Abstract

Sm^3+^ and Ce^3+^ singly doped and Sm^3+^ and Ce^3+^ co-doped Sr_3_B_2_O_6_ phosphors are prepared via a high-temperature solid-state reaction method. The crystal structure and phase purity are characterized by X-ray diffraction (XRD) analyses. The Sm^3+^-doped sample displays an emission in the orange-red region, with the strongest emission line at about 648 nm and possessing a good luminescence thermal stability between 78 and 500 K. With the increase in the Sm^3+^ content, the concentration quenching is observed due to the cross-relaxation (CR) processes among the Sm^3+^ ions. Upon 340 nm excitation, the Ce^3+^-doped phosphor presents a broad emission band in the blue region with a maximum at about 420 nm, which overlaps well with the ^6^H_5/2_ → ^6^P_3/2_ excitation line of Sm^3+^ and implies the possible energy transfer from Ce^3+^ to Sm^3+^. The spectral and decay measurements of the Ce^3+^ and Sm^3+^ co-doped samples are conducted and the Inokuti–Hirayama (I-H) model is adopted to analyze the luminescence decay dynamics of the donor Ce^3+^. Owing to the evident sensitization of the Sm^3+^ by the Ce^3+^ ions, the co-doped samples exhibit color variation under different wavelength excitations, endowing them with potential applications in optical anti-counterfeiting.

## 1. Introduction

Lanthanide-activated luminescence materials with stimuli-responsive emission outputs have attracted increasing attention due to their potential applications in optical anti-counterfeiting and information security fields [1,2,3,4,5,6]. To pursue the superior performances of feasibility and reliability for applications, there is a necessity for the constant exploration of novel phosphors with an easy trigger and an abundant color evolution. Recently, the excitation-wavelength-responsive lanthanide-activated phosphors including upconversion [1] and downshifted phosphors [2] have emerged as important alternatives owing to the feature of a convenient excitation trigger [7,8], when compared to other stimuli methods such as temperature [9], pressure [10], and so on. With the merit of high emission efficiency, the downshifted lanthanide-activated phosphors significantly promote the performances of optical anti-counterfeiting and information security applications. To further expand their color evolution range with the trigger of excitation wavelength, a feasible way is by simply co-doping two or more different lanthanide ions with distinct emission colors into a suitable host compound. Thus, the emission color and the excitation wavelength may be tuned and expanded by virtue of the possible energy transfer among these lanthanide ions.

Sm^3+^ ions usually display strong 4f-4f absorption lines at about 400 nm in most compounds, which match well with the commercially available light-emitting diode (LED) chips. Meanwhile, Sm^3+^-doped phosphors usually exhibit an intense emission in the orange-red region. Therefore, it is easy to achieve monochromatic anti-counterfeiting with a commercial 400 nm LED chip by choosing Sm^3+^ ions as activators.

In addition, the energy of the parity-allowed 4f-5d transition of Ce^3+^ is evidently affected by the host crystal field and electron–phonon coupling, due to its outer-lying 5d orbitals. The emission of Ce^3+^ is found to vary from UV to the red region [11,12,13] in different types of host compounds. It can be deduced that if Ce^3+^ ions display a blue-violet emission around 400 nm in a well-selected host compound, then Ce^3+^–Sm^3+^ energy transfer (ET) may occur, and it is possible to realize a wide range of luminescence tuning from a blue-violet emission of Ce^3+^ to an orange-red emission of Sm^3+^ under different excitation conditions, by utilizing their different emission colors and the potential ET effect.

Based on the above considerations, we chose Sr_3_B_2_O_6_ (abbreviated as SBO hereafter) as a host compound in this paper due to its simple synthesis condition, good physicochemical stability [14] and one kind of Sr^2+^ site for lanthanide occupation. The influence of temperature and concentration on Sm^3+^ luminescence and the energy transfer dynamics between the Ce^3+^ and the Sm^3+^ in the SBO host were studied. The results reveal the emission color-tunability of the co-doped samples upon different excitation wavelengths and demonstrate their potential applications in optical anti-counterfeiting. This work provides a simple strategy and a candidate material towards excitation-wavelength-responsive optical anti-counterfeiting.

## 2. Materials and Methods

A series of Sm^3+^ and Ce^3+^ singly doped (Sr_3−2x_Sm_x_Na_x_B_2_O_6_, x = 0.005, 0.01, 0.02, 0.03, 0.05, 0.10, SBO:xSm^3+^; Sr_2.98_Ce_0.01_Na_0.01_B_2_O_6_, SBO:0.01Ce^3+^) and Ce^3+^ and Sm^3+^ co-doped samples (Sr_2.98−2y_Ce_0.01_Sm_y_Na_0.01+y_B_2_O_6_, y = 0.005, 0.01, 0.02, 0.03, 0.05, 0.10, SBO:0.01Ce^3+^, ySm^3+^) were prepared via a high-temperature solid-state reaction method using SrCO_3_ (Analytical Reagent, A. R.), H_3_BO_3_ (A. R.), Na_2_CO_3_ (A. R.), Sm_2_O_3_ (99.99%) and/or CeO_2_ (99.99%) with stoichiometric ratios as reactants. The raw materials were fully ground in agate mortars, then transferred into corundum crucibles and heated at 1423 K for 6 h in a reductive H_2_-N_2_ (5–95%) ambience. After cooling down to room temperature (RT) naturally in the furnace, the products were re-ground for further characterizations.

The phase purity of the powder samples was checked by X-ray diffraction (XRD) measurements on a RIGAKU D-MAX 2200 VPC X-ray diffractometer (Tokyo, Japan) with Cu Kα radiation (λ = 1.5418 Å) at an operating condition of 40 kV and 26 mA. The XRD data were collected over a 2*θ* range of 10–80° with a scanning rate of 10°/min. The high-quality XRD data of the SBO:0.005Sm^3+^ for Rietveld refinement were recorded over a 2*θ* range from 10° to 110° at an interval of 0.02° using a Bruker D8 ADVANCE X-ray diffractometer (Billerica, MA, USA) with Cu Kα radiation (λ = 1.5418 Å) at 35 kV and 35 mA. Rietveld refinement was performed with the *TOPAS*-academic *V*4.1 program [15]. The morphology of the SBO:0.005Sm^3+^ sample was characterized with an FEI Quanta 400 FEG scanning electron microscope (SEM) (Lincoln, NE, USA). The photoluminescence (PL) spectra and luminescence decay curves at RT and varied temperatures were measured on an Edinburgh FLS 1000 combined spectrometer equipped with a PMT detector (Livingston, UK). A 450 W Xe900 xenon lamp was used as an excitation source of steady-state PL measurements; a 340 nm pulsed light-emitting diode (EPLED, Edinburgh Instruments) was used to collect the luminescence decay curves of Ce^3+^, while a 60 W μF900 flash lamp with a pulse width of 1.5–3.0 μs and a pulse repetition rate of 20 Hz was used to record the luminescence decay curves of the Sm^3+^ in a microsecond range. The temperature-dependent luminescence measurements in 78–500 K were performed with an Oxford Optistat DN cryostat (Abingdon, UK), and the sample temperature was controlled by a MercuryiTC temperature controller. Sample images upon UV excitation were taken with an Apple iPhone 8 Plus mobile phone at a normal condition.

## 3. Results and Discussion

### 3.1. Crystal Structure, Morphology and Phase Purity

**Figure 1:**
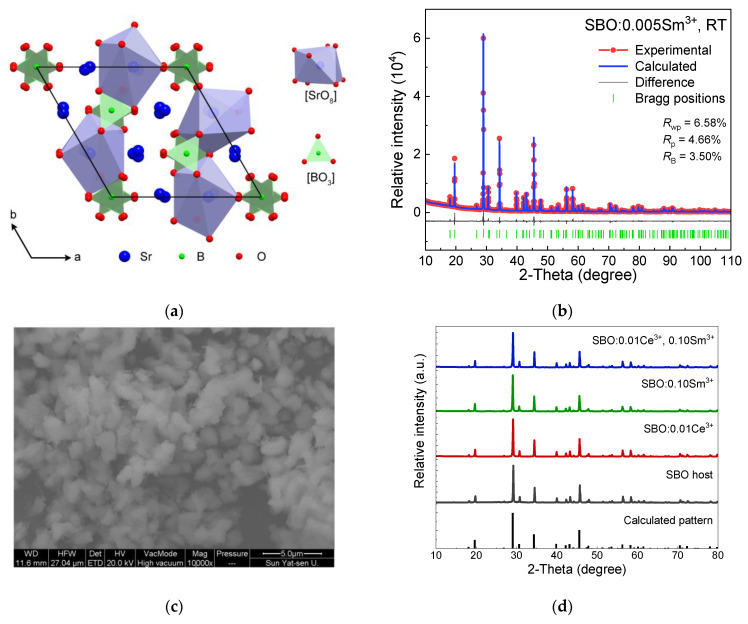
(**a**) Crystal structure along *c*-axis of Sr_3_B_2_O_6_ host as well as [SrO_8_] polyhedron and [BO_3_] triangle; (**b**) Rietveld refinement of SBO:0.005Sm^3+^ XRD data at RT; (**c**) Scanning electron microscope (SEM) image of SBO:0.005Sm^3+^ sample; (**d**) XRD patterns of some typical samples.

The SBO crystallizes in a trigonal system with a space group of *R*-3*c* (No. 167) [16]. There is only one kind of Sr^2+^ site in the SBO, which coordinates with eight oxygen ions to form a dodecahedron geometry with *C*_2_ symmetry, as shown in Figure 1a. Each B^3+^ ion coordinates with three oxygen ions to form a [BO_3_]^3−^ planar triangle. Due to the similar ion radii of Sr^2+^ and lanthanide dopants, as well as the charge compensator Na^+^ (coordination number, C. N. = 8, *r*(Sr^2+^) = ~1.26 Å, *r*(Ce^3+^) = ~1.14 Å, *r*(Sm^3+^) = ~1.08 Å, *r*(Na^+^) = ~1.18 Å) [17], the Ce^3+^, Sm^3+^ and Na^+^ ions are expected to occupy the Sr^2+^ sites. Rietveld refinement of the SBO:0.005Sm^3+^ sample is performed and shown in Figure 1b. The values of *R*_p_ (~6.58%), *R*_wp_ (~4.66%) and *R*_B_ (~3.50%) indicate a reliable refinement result. The refined lattice parameters are *a* = *b* = 9.044(1) Å, *c* = 12.572(1) Å, *γ* = 120°, *V* = 890.6(1) Å^3^ and *Z* = 6, and other related parameters are listed in Table 1. The average Sr^2+^/Sm^3+^-O^2−^ bond length is about 2.634(4) Å, while the adjacent distance of the nearest Sr^2+^ ions is evaluated at about 3.594 Å. Figure 1c display the SEM image of the SBO:0.005Sm^3+^ sample. Agglomerate particles with irregular shapes and an average size of several microns are observed. Some representative XRD patterns of the host compound and the singly or doubly doped samples are displayed in Figure 1d. The patterns of the un-doped host and doped samples match well with that of the theoretical result calculated with the Crystallographic Information File (CIF) of the SBO [16]. Furthermore, the lattice parameters of the representative samples are also calculated via Rietveld refinement as tabulated in Table 2. They clearly show the lattice shrinkages with different degrees, which depends on the doping ionic size and concentration. These results imply the successful doping of lanthanides into the SBO host lattice.

### 3.2. Luminescence Properties of Sm^3+^ in SBO Host

**Figure 2:**
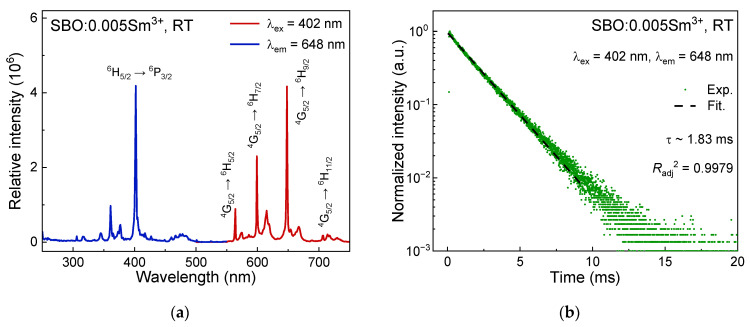

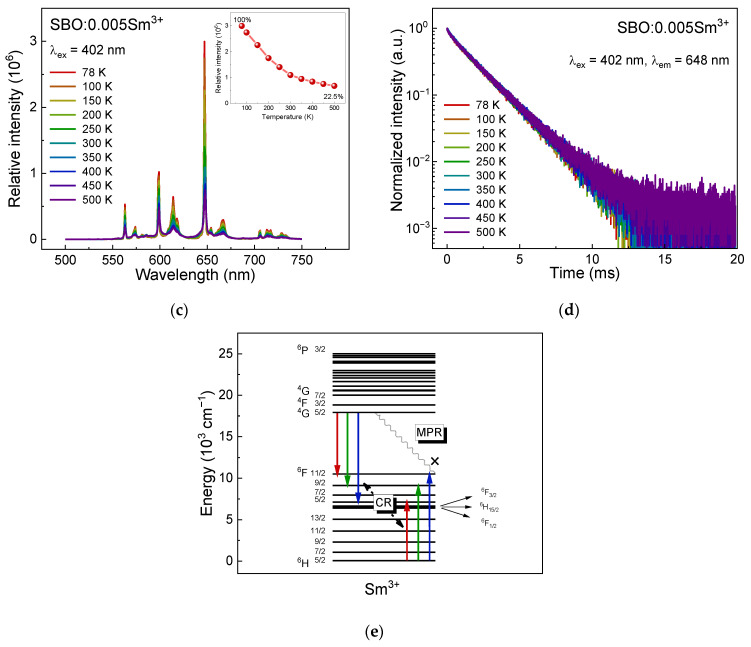
(**a**) Excitation (λ_em_ = 648 nm) and emission spectra (λ_ex_ = 402 nm) of SBO:0.005Sm^3+^ at RT; (**b**) Luminescence decay curve (λ_ex_ = 402 nm, λ_em_ = 648 nm) of SBO:0.005Sm^3+^ at RT; (**c**) Temperature-dependent emission spectra (λ_ex_ = 402 nm) of SBO:0.005Sm^3+^ from 78 to 500 K; (**d**) Temperature-dependent decay curves (λ_ex_ = 402 nm, λ_em_ = 648 nm) of SBO:0.005Sm^3+^; (**e**) Partial energy levels and non-radiative processes of Sm^3+^.

Figure 2a displays the excitation and emission spectra of the SBO:0.005Sm^3+^ at RT. When monitoring the emission wavelength at 648 nm, some sharp line-like excitation peaks can be observed in the range of 250–480 nm, which are attributed to the transitions from the ^6^H_5/2_ ground state to higher 4f levels of Sm^3+^. The dominant peak at about 402 nm is assigned to the ^6^H_5/2_ → ^6^P_3/2_ transition of the Sm^3+^ ion [18]. Upon 402 nm excitation, one can identify about three relatively intensive sharp peaks in the emission spectra in Figure 2a, which can be aligned as ^4^G_5/2_ → ^6^H_J_ transitions of the Sm^3+^ for J = 5/2 (~564 nm), 7/2 (~599 nm) and 9/2 (~648 nm), respectively [19]. The strongest emission peak of the Sm^3+^ in our case is located at 648 nm originating from the ^4^G_5/2_ → ^6^H_9/2_ electric–dipole transition, similar to the results in YVO_4_ [20,21], Sr_2_CaMoO_6_ [22] and Ca_2_GdNbO_6_ [23] with asymmetric cationic environments, but is different with some cases where the Sm^3+^ sits at high-symmetrical sites [24,25,26]. The luminescence of the SBO:0.005Sm^3+^ displays exponential decay with a lifetime of about 1.83 ms, as shown in Figure 2b.

In order to study the temperature-related luminescence properties of Sm^3+^, we measured the emission spectra as well as the decay curves of the SBO:0.005Sm^3+^ at 78–500 K as given in Figure 2c,d. The spectral profiles at different temperatures nearly remain identical, although a slight thermal broadening is observed. The peak intensity (relative height) at 648 nm in the inset of Figure 2c shows a decline from 78 to 500 K, where the peak intensity at 500 K remains at 22.5% of that at 78 K. However, the overlapped exponential decay curves in Figure 2d manifest the good thermal stability of the Sm^3+^ 4f-4f luminescence. The discrepancy between the results obtained from luminescence intensity and luminescence decay measurements is mainly due to the complicated factors attributed to the spectral measurements, such as the temperature-dependence of absorption strength, energy migration and reabsorption [27]. Decay measurement seems to provide a more reasonable insight into the thermal-quenching behavior. The thermal quenching of Sm^3+^ is mainly related to the multi-phonon relaxation (MPR) process [28] where several phonons synergistically depopulate the higher-lying excited electrons of the Sm^3+^, as depicted in Figure 2e. The MPR process of Sm^3+^ depends on the energy gap (Δ*E*) between ^4^G_5/2_ and ^6^F_11/2_ (~7400 cm^−1^), as well as the effective phonon energy (ℏω ~ 900 cm^−1^ in Sr_3_B_2_O_6_ in [29]. The number of phonons (*p*) is derived by *p* = Δ*E*/ℏω. The good thermal stability of Sm^3+^ in our case indicates the ineffective MPR process that requires too many phonons (~8) to bridge the energy gap between the two energy levels.

**Figure 3:**
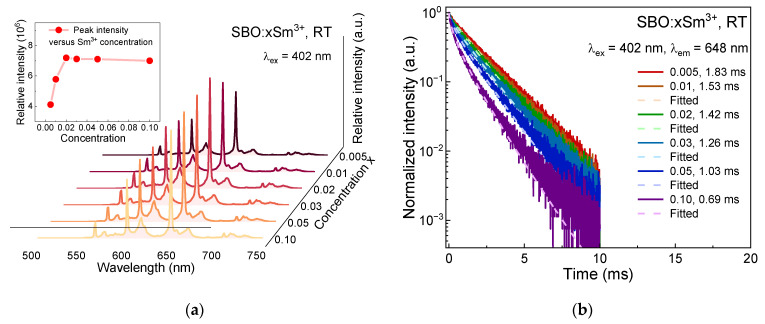
(**a**) Concentration-dependent emission spectra (λ_ex_ = 402 nm) of SBO:0.005Sm^3+^ at RT, the inset shows the peak intensity at 648 nm as a function of doping concentration; (**b**) The corresponding concentration-dependent decay curves (λ_ex_ = 402 nm, λ_em_ = 648 nm) and fitted results via Inokuti-Hirayama (I-H) model of SBO:xSm^3+^ (x = 0.005, 0.01, 0.02, 0.03, 0.05, 0.10) at RT.

The concentration-dependent emission spectra of the SBO:xSm^3+^ (x = 0.005, 0.01, 0.02, 0.03, 0.05, 0.10) are presented in Figure 3a. The emission profile does not change with increasing dopants, while the intensity comes to a maximum when x = 0.02 and slightly decreases above this concentration as shown in the inset. The luminescence decay curves of the SBO:xSm^3+^ are also measured and shown in Figure 3b. At a low concentration (x = 0.005, 0.01), the decay curves stay mono-exponential but they deviate gradually from the exponential form at higher concentrations, indicating that concentration quenching happens in the concentrated samples. The average lifetime values of the samples are then obtained via the following equation [30]:

(1) $$\tau =\int_{0}^{\infty }\frac{I\left(t\right)}{I\left(0\right)}dt$$

where *I*(0) and *I*(*t*) denote the luminescence intensity when time is 0 and *t*, respectively. The average lifetime values are listed in the legend of Figure 3b and are shortened with the increase of the Sm^3+^ concentration. When dopants increase, the Sm^3+^-Sm^3+^ interaction distance is shortened, and the dense energy levels of the Sm^3+^ endow them with some resonant non-radiative channels to quench the luminescence of the Sm^3+^ via a cross-relaxation (CR) process. Some CR channels of the Sm^3+^, such as [^4^G_5/2_, ^6^H_5/2_] → [^6^F_11/2_, ^6^F_5/2_], [^4^G_5/2_, ^6^H_5/2_] → [^6^F_5/2_, ^6^F_11/2_], [^4^G_5/2_, ^6^H_5/2_] → [^6^F_9/2_, ^6^F_9/2_], and so on, have been reported and schematized in Figure 2e [22,24,31,32].

To further study the CR mechanism of Sm^3+^ in the SBO host, we applied the Inokuti–Hirayama (I-H) model to analyze the luminescence decay curves of the Sm^3+^-doped samples as shown in the following equation [33]:

(2) $$\frac{I\left(t\right)}{I\left(0\right)}=exp\left[-\left(\frac{t}{\tau_{0}}\right)-\frac{4\pi }{3}C_{A}\Gamma \left(1-\frac{3}{S}\right){\left(C_{\mathrm{DA}}\right)}^{3/S}t^{3/S}\right]$$

where *I*(*t*) denotes the intensity at a given time *t*, *I*(0) is the initial intensity, *τ*_0_ is the intrinsic lifetime of Sm^3+^ (~1.83 ms), and *C*_A_ is doping concentration of Sm^3+^. *C*_DA_ is the ET microparameter. *Γ*(x) is the gamma function and *S* expresses the multipolar interaction (6 for electric dipole-dipole (EDD) interaction, 8 for electric dipole-quadrupole (EDQ) and 10 for electric quadrupole-quadrupole (EQQ)). The energy transfer probability *P*_DA_ can be expressed with the following equation:

(3) $$P_{\mathrm{DA}}=C_{\mathrm{DA}}^{\left(S\right)}/R^{S}$$

where *R* is denoted as the distance between ions. When *P*_DA_ = 1/*τ*_0_, where the ET rate of donor ion equals to its own radiation rate, the obtained *R* is defined as ET critical distance (*R*_c_). The fitted results are compiled in Table 3. When *S* is set to be 6, the best fitting quality can be obtained, indicating that the main interaction type of CR in Sm^3+^ ions is EDD interaction, similar to the result in [34,35]. The averaged *C*_DA_ value is ~3.730 × 10^−53^ m^6^/s, and the corresponding *R*_c_ is calculated to be about 6.40 Å.

### 3.3. Luminescence Properties of SBO:0.01Ce^3+^ Sample

**Figure 4:**
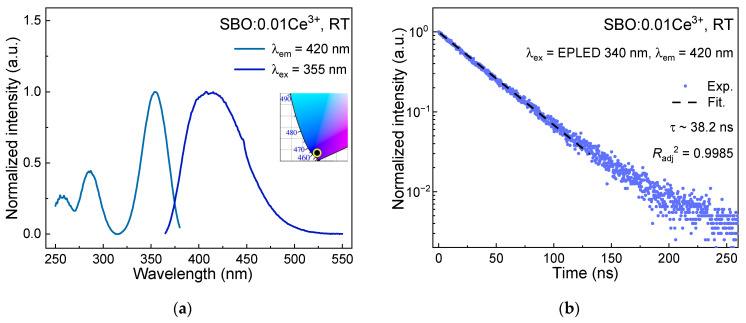
(**a**) The highest-height normalized excitation (λ_em_ = 420 nm) and emission (λ_ex_ = 340 nm) spectra of SBO:0.01Ce^3+^ at RT. The inset displays the CIE chromaticity coordinates of the sample; (**b**) Luminescence decay curve (λ_ex_ = EPLED 340 nm, λ_em_ = 420 nm) of SBO:0.01Ce^3+^ at RT.

Figure 4a shows the highest-height normalized excitation and emission spectra of a diluted sample SBO:0.01Ce^3+^ at RT. Monitoring 420 nm emission, one can observe three broad bands over a range of 250–380 nm, which are assigned as the transitions from 4f ground state to 5d_i_ (i = 1, 2, 3) excited states of Ce^3+^ in descending order in wavelength. Upon 340 nm excitation, Ce^3+^ exhibits a broad emission band with a maximum at ~420 nm, consistent with the reported result in [36,37]. The Commission Internationale de l’Eclairage (CIE) chromaticity coordinates are calculated as (0.157, 0.031), lying in the blue-violet region as shown in the inset of Figure 4a. The luminescence decay curve in Figure 4b displays an exponential feature, and the lifetime of the Ce^3+^ emission is fitted to be ~38.2 ns.

### 3.4. Luminescence of Sm^3+^, Ce^3+^ Co-Doped Samples and Ce^3+^-Sm^3+^ ET Dynamics

**Figure 5:**
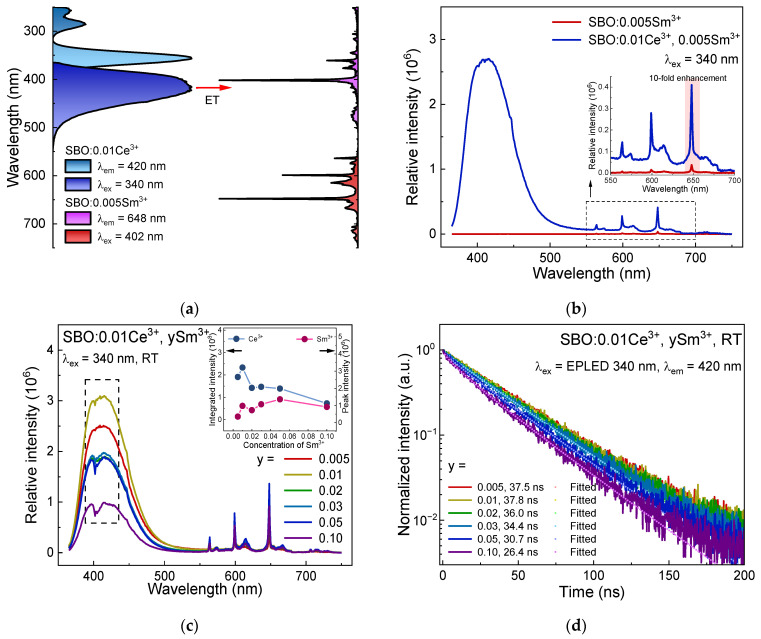
(**a**) Normalized excitation (λ_em_ = 420 for Ce^3+^, 648 nm for Sm^3+^) and emission spectra (λ_ex_ = 340 for Ce^3+^, 402 nm for Sm^3+^) of SBO:0.01Ce^3+^ and SBO:0.005Sm^3+^ at RT; (**b**) Emission spectra (λ_ex_ = 340 nm) of SBO:0.01Ce^3+^ and SBO:0.01Ce^3+^, 0.005Sm^3+^ at RT; (**c**) Emission spectra (λ_ex_ = 340 nm) of SBO:0.01Ce^3+^, ySm^3+^ (y = 0.005, 0.01, 0.02, 0.03, 0.05, 0.10) at RT, the inset display the integrated intensity of Ce^3+^ emission and peak intensity of Sm^3+^ emission as a function of y; (**d**) Luminescence decay curves (λ_ex_ = EPLED 340 nm, λ_em_ = 420 nm) of SBO:0.01Ce^3+^, ySm^3+^ (y = 0.005, 0.01, 0.02, 0.03, 0.05, 0.10) and corresponding fitted results via I-H model.

Figure 5a shows the highest-height normalized excitation and emission spectra of SBO:0.01Ce^3+^ and SBO:0.005Sm^3+^ at RT. A spectral overlap of the Ce^3+^ emission and the Sm^3+^ excitation over a range of 365–550 nm can be observed, suggesting the possibility of ET from Ce^3+^ to Sm^3+^. Figure 5b displays the spectral difference of the Sm^3+^ singly doped and the Ce^3+^ and Sm^3+^ co-doped sample. Upon excitation at 340 nm where Ce^3+^ can be efficiently excited but the absorption of Sm^3+^ is very weak, the Sm^3+^ singly doped sample has a similar emission spectrum, but an evidently weaker signal compared to that in Figure 2a. In the Ce^3+^ and Sm^3+^ co-doped case, both the emission of Ce^3+^ and Sm^3+^ can be observed. Under 340 nm excitation, the intensity of Sm^3+^ shows a 10-fold enhancement compared to the singly doped one, indicating the ET from Ce^3+^ to Sm^3+^ greatly sensitizes the luminescence of Sm^3+^.

When further increasing the Sm^3+^ concentration in the SBO:0.01Ce^3+^, ySm^3+^ (y = 0.005–0.10) samples, Ce^3+^ emission decreases with increasing y values except for the outlier SBO:0.01Ce^3+^, 0.01Sm^3+^, which may be due to an experimental error, as shown in Figure 5c. One can also notice that there is a V-shaped notch in the Ce^3+^ emission spectra at 402 nm, as marked with a dashed rectangle because of the absorption of Sm^3+^ [18]. The reduction in Ce^3+^ intensity is mainly due to the ET from Ce^3+^ to Sm^3+^. This can be also confirmed by the Ce^3+^ decay curves in Figure 5d after ruling out the concentration quenching of Ce^3+^ in the SBO host, which was studied in our previous work [37].

The Sm^3+^ emission intensity in Figure 5c shows an increase when y = 0.005–0.05, and then decreases. Two opposite effects, the Ce^3+^-Sm^3+^ ET and the Sm^3+^-Sm^3+^ CR, jointly affect the luminescence intensity of Sm^3+^ in the co-doped samples. The ET enhances the Sm^3+^ luminescence while the CR weakens it. At low concentrations, the ET effect plays a more important role, so the Sm^3+^ emission intensity increases with the increasing doping contents. Then, the CR becomes dominant in highly-doped samples where the ET seems insufficient to offset the CR, thus leading to a decline in Sm^3+^ emission. The luminescence decay curves of Ce^3+^ in co-doped samples (Figure 5d) gradually deviate from exponential, and their average lifetime values obtained via Equation (1) are also shortened from 37.5 ns (y = 0.005) to 26.4 ns (y = 0.10) (see the legends in Figure 5d), indicating that the ET from Ce^3+^ to Sm^3+^ accelerates the depopulation process of the Ce^3+^ excited electrons. The I-H model is also utilized to analyze the decay properties of the Ce^3+^. The fitted result and relevant parameters are displayed in Figure 5d and Table 4. When *S* is set to be 6, we obtain the best fitting, showing that the main multipolar interaction in the Ce^3+^-Sm^3+^ ET process is EDD type [38,39]. The average *C*_DA_ in the Ce^3+^-Sm^3+^ ET process is ~2.268 × 10^−49^ m^6^/s. 

### 3.5. Color Variation under Different Excitation Conditions of Samples and Their Potential Anti-Counterfeiting Applications

**Figure 6:**
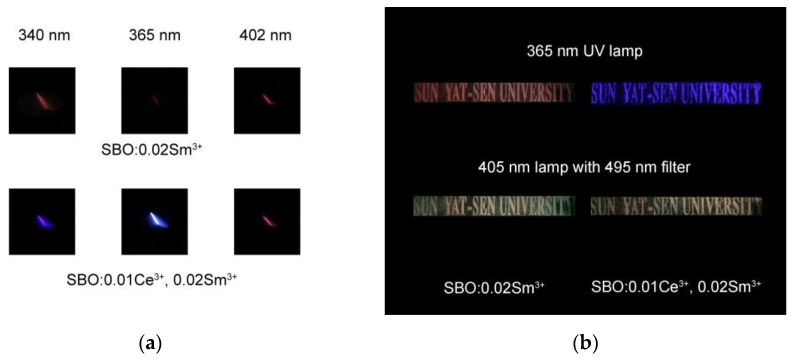
(**a**) Luminescent images of SBO:0.02Sm^3+^ and SBO:0.01Ce^3+^, 0.02Sm^3+^ upon 340, 365 and 402 nm excitation of xenon lamp at RT; (**b**) Luminescent patterns of “SUN YAT-SEN UNIVERSITY” under 365 nm and 405 nm commercial lamp excitations based on SBO:0.02Sm^3+^ and SBO:0.01Ce^3+^, 0.02Sm^3+^ samples.

With the distinguished emission colors of the Ce^3+^ and the Sm^3+^ as well as their ET effect, color variation under different excitation wavelengths in the co-doped samples can be anticipated. Figure 6a exhibits luminescent images of the SBO:0.02Sm^3+^ and the SBO:0.01Ce^3+^, 0.02Sm^3+^ upon 340, 365 and 402 nm excitation of xenon lamp at RT. Monochromatic emission from Sm^3+^ is observed in the SBO:0.02Sm^3+^ sample with different excitation wavelengths. For the SBO:0.01Ce^3+^,0.02Sm^3+^ sample, one can observe a color evolution from violet-blue, sky-blue to orange-reddish color under 340, 365 and 402 nm excitation, respectively. At 340 and 365 nm excitation, Ce^3+^ can be efficiently excited and the Sm^3+^ orange-reddish emission can be greatly enhanced in virtue of the ET. Hence, the sample display is a mixed color of Ce^3+^ and Sm^3+^. When exciting at 402 nm where Sm^3+^ has a strong excitation peak but Ce^3+^ shows no absorption, only the characteristic red emission of Sm^3+^ is observed. The color tunability of the co-doped samples upon different excitation conditions may be applied in optical anti-counterfeiting. Figure 6b shows the anti-counterfeiting application demonstration using the SBO:0.02Sm^3+^ and the SBO:0.01Ce^3+^, 0.02Sm^3+^ samples. A patterned quartz glass substrate was first prepared via a laser printing and corrosion procedure, as introduced in our previous works [40,41]. The corresponding sample powders are filled into the substrate to form the pattern. Upon 365 nm commercial UV lamp excitation, the two samples display a red and violet-blue pattern of “SUN YAT-SEN UNIVERSITY”, respectively, resembling the result in Figure 6a. Furthermore, when we used a commercial 405 nm LED lamp and a 495 nm filter to eliminate the intensive excitation light, and both the two samples exhibited an orange-reddish pattern originating from the Sm^3+^ luminescence. These results manifest their potential applications in optical anti-counterfeiting.

## 4. Conclusions

The Sm^3+^ and Ce^3+^ singly doped and Sm^3+^ and Ce^3+^ co-doped SBO samples were prepared via a conventional high-temperature solid-state reaction method. XRD patterns of the doped samples reveal their single phase of the SBO. Upon 402 nm excitation, the Sm^3+^ singly doped sample exhibits an orange-reddish emission at about 648 nm, which is assigned as the ^4^G_5/2_ → ^6^F_9/2_ transition and possesses a radiative lifetime of ~1.83 ms. The Sm^3+^ luminescence maintains good thermal stability up to 500 K with identical temperature-dependent decay curves. Concentration quenching occurs in samples with high dopants, which is due to the CR process of Sm^3+^ ions. I-H model analysis reveals that the main mechanism of the CR is EDD interaction with *C*_DA_ ~3.730 × 10^−53^ m^6^/s and a critical interaction distance of ~6.40 Å. The Ce^3+^ doped sample SBO:0.01Ce^3+^ displays a broad emission band in the blue region at ~420 nm with a lifetime of ~38.2 ns. A spectral overlap between Ce^3+^ emission and Sm^3+^ excitation is observed, manifesting a possible ET from Ce^3+^ to Sm^3+^. Upon 340 nm of the Ce^3+^ 4f-5d transition in the co-doped samples, the Sm^3+^ luminescence is remarkably enhanced, and the decay curves of the Ce^3+^ in co-doped samples deviate from exponential features, which verify the Ce^3+^-Sm^3+^ ET. The I-H analysis on decay curves of the Ce^3+^ luminescence indicates the main EDD interaction type in the ET process from Ce^3+^ to Sm^3+^. With tunable luminescence upon different wavelength conditions, the Ce^3+^ and Sm^3+^ co-doped SBO samples are regarded as promising candidates for optical anti-counterfeiting.

## Figures and Tables

**Table 1:** Refined parameters of SBO:0.005Sm^3+^ sample.

Atom	x	y	z	Occ.	*B*_iso_
Sr	0.3554(1)	0	0.2500	0.9966	1.1227(13)
Sm	0.3554(1)	0	0.2500	0.0017	1.3435(13)
Na	0.3554(1)	0	0.2500	0.0017	0.9375(21)
B	0	0	0.1185(8)	1	1.3979(20)
O	0.1601(4)	0.0118(5)	0.1156(2)	1	1.1704(42)

**Table 2:** Calculated lattice parameters of doped samples.

Samples	SBO:0.01Ce^3+^	SBO:0.10Sm^3+^	SBO:0.01Ce^3+^, 0.10Sm^3+^
*a* (Å)	9.042(1)	9.039(2)	9.038(2)
*c* (Å)	12.570(3)	12.567(3)	12.565(3)
*V* (Å^3^)	890.3(2)	889.5(1)	889.2(3)

**Table 3:** Relevant parameters in I-H model analysis.

Sm^3+^ Concentration	*C*_A_ (×10^25^ m^−3^)	*C*_DA_ (×10^−53^ m^6^/s)	*R*_adj_^2^
0.01	6.742	2.740	0.9975
0.02	13.48	3.338	0.9978
0.03	20.23	3.856	0.9974
0.05	33.71	4.542	0.9973
0.10	67.42	4.175	0.9973
average	-	3.730	-

**Table 4:** Relevant parameters in I-H model analysis in Ce^3+^ and Sm^3+^ co-doped samples.

Sm^3+^ Concentration	*C*_A_ (×10^25^ m^−3^)	*C*_DA_ (×10^−49^ m^6^/s)	*R*_adj_^2^
0.005	3.371	0.8791	0.9978
0.01	6.742	2.424	0.9983
0.02	13.48	1.393	0.9983
0.03	20.23	2.359	0.9978
0.05	33.71	4.089	0.9984
0.10	67.42	2.464	0.9982
average	-	2.268	-

## Data Availability

Data sharing is not applicable to this article.
